# Autonomous Navigation of a Center-Articulated and Hydrostatic Transmission Rover using a Modified Pure Pursuit Algorithm in a Cotton Field

**DOI:** 10.3390/s20164412

**Published:** 2020-08-07

**Authors:** Kadeghe Fue, Wesley Porter, Edward Barnes, Changying Li, Glen Rains

**Affiliations:** 1 College of Engineering, University of Georgia, Athens, GA 30602, USA; cyli@uga.edu; 2 Department of Entomology, University of Georgia, Tifton, GA 31793, USA; 3 Department of Crop and Soil Sciences, University of Georgia, Tifton, GA 31793, USA; wporter@uga.edu; 4 Cotton Incorporated, Cary, NC 27513, USA; ebarnes@cottoninc.com

**Keywords:** robotics, GNSS, cotton robotics, autonomous navigation, RTK, ROS

## Abstract

This study proposes an algorithm that controls an autonomous, multi-purpose, center-articulated hydrostatic transmission rover to navigate along crop rows. This multi-purpose rover (MPR) is being developed to harvest undefoliated cotton to expand the harvest window to up to 50 days. The rover would harvest cotton in teams by performing several passes as the bolls become ready to harvest. We propose that a small robot could make cotton production more profitable for farmers and more accessible to owners of smaller plots of land who cannot afford large tractors and harvesting equipment. The rover was localized with a low-cost Real-Time Kinematic Global Navigation Satellite System (RTK-GNSS), encoders, and Inertial Measurement Unit (IMU)s for heading. Robot Operating System (ROS)-based software was developed to harness the sensor information, localize the rover, and execute path following controls. To test the localization and modified pure-pursuit path-following controls, first, GNSS waypoints were obtained by manually steering the rover over the rows followed by the rover autonomously driving over the rows. The results showed that the robot achieved a mean absolute error (MAE) of 0.04 m, 0.06 m, and 0.09 m for the first, second and third passes of the experiment, respectively. The robot achieved an MAE of 0.06 m. When turning at the end of the row, the MAE from the RTK-GNSS-generated path was 0.24 m. The turning errors were acceptable for the open field at the end of the row. Errors while driving down the row did damage the plants by moving close to the plants’ stems, and these errors likely would not impede operations designed for the MPR. Therefore, the designed rover and control algorithms are good and can be used for cotton harvesting operations.

## 1. Introduction

Mechanical harvesting has helped improve crop production significantly since the mid-1900s. Before these machines were developed, crops such as cotton were primarily hand-harvested. The development of the cotton combine helped to reduce labor costs and increase production efficiency but comes with its downsides. Harvesting is accomplished using expensive machines that are massive in size and weight, which can lead to soil compaction, and are also costly and time-consuming to repair. Breakdowns during the season may expose cotton to hostile environmental conditions that can diminish the quality of the harvest. Additionally, cotton harvesting takes place only after cotton fields have been defoliated with chemical defoliants. These chemicals can degrade the land and are an added expense to production costs. The defoliation is performed approximately 50 days from the opening of the first bolls. A consequence of waiting many weeks to harvest all the cotton simultaneously, lint quality is affected profoundly by external weather and other environmental elements from the time they open to the time they are picked [1,2]. As a consequence of the current cotton management system, small acreage farmers can not afford to buy these machines or maintain them [3,4]. Furthermore, the fast aging farming community will experience a labor shortage, since many of their children are moving to urban areas [5].

Any solution that could increase the participation of small and family farmers would be well received. One alternative to current large-scale farm and machinery systems is to introduce small, multi-purpose, robotic rovers that can navigate and perform agricultural operations autonomously without the need for human–machine control. Fortunately, there is a booming industry in robotics and machine learning technologies, and robotics have been developed to solve many pertinent issues in agriculture [1,2,5,6,7].

**Figure 1:**
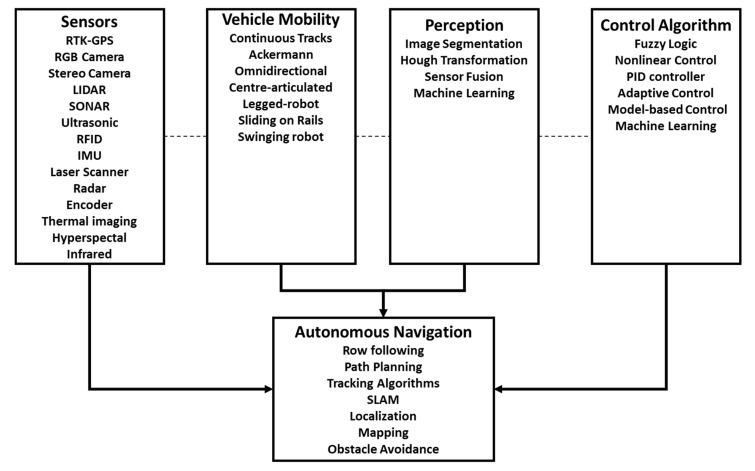
Autonomous navigation modules.

However, for mobile robotic systems to be efficient, they need very effective methods to navigate fields autonomously. The autonomous navigation of robotic systems depends upon four modules: sensors, vehicle mobility, perception, and control algorithms (Figure 1). Sensors, such as RTK-GNSS, Red-Green-Blue (RGB) cameras, Stereo camera, Light Detection and Ranging (LiDAR), Sound Navigation and Ranging (SONAR), Ultrasonic, Radio-frequency identification (RFID), Inertial Measurement Unit (IMU), Laser scanner, RAdio Detection And Ranging (RADAR), encoders, thermal imaging, hyperspectral, and infrared, have been used extensively to detect fruits and plants in agricultural fields (Figure 1) [5,7,8,9,10,11,12,13,14,15,16,17,18,19,20,21,22,23,24,25]. Additionally, many vehicle mobility systems have been developed primarily for agricultural use, such as continuous tracks, the Ackermann four-wheel drive, center-articulated drives, legged-robots, swinging robots, omnidirectional drives, and sliding-on-the-rail robots (Figure 1). The mobility is designed to accommodate different soil and topographic conditions, open or greenhouse farming, and maneuverability requirements. Perception is created using sensor output and machine vision algorithms, such as image segmentation, hough transformation, sensor fusion, or machine learning, to obtain environmental features (Figure 1). Models that can do perception with the lower cost of computation are essential to be used in small mobile robots [26,27]. After perceiving the environment, the mobile robot performs a movement action to the next location. There are multiple control system methods used: fuzzy logic, nonlinear, proportional-integral-derivative (PID), adaptive, model-based, rear-wheel feedback, linear-quadratic regulator, model predictive control (MPC), and machine learning, such as neural networks and reinforcement learning. After assimilating a control system, several techniques must be incorporated to develop autonomous navigation in an unstructured environment, such as localization, mapping, obstacle avoidance, simultaneous localization, and Mapping (SLAM), row-following in row crops and path planning to perform complex farm operation movements [5,7,8,9,10,11,12,13,14,15,16,17,18,19,20,21,22,23,24,25]. Furthermore, algorithms that guide the robots to avoid obstacles should be incorporated so that the robot can work smoothly in the agricultural field while interacting with humans and other robots [28,29,30]. Additionally, there are proposals to use the Internet of Things and sensors to monitor and detect the operating conditions of vehicles, such as fluid leaking in a hydrostatic tractor, which can be incorporated in robotic systems too [31,32]. Additionally, all the computations to guide the robot can be done onboard to reduce network traffic and develop green communications [33]. Robot navigation in field crops has been studied well in electric vehicles with Ackerman and omnidirectional steering [34,35]. Limited work has been put forward for the center-articulated vehicle, which is a special kind of off-road vehicle that has very high traction and maneuverability and, hence, is common in agriculture, forestry, and construction industry [36]. Center-articulated vehicles can easily turn corners in a constrained environment by using two hydraulic cylinders that can change the yaw angle of the two parts of the vehicle [36]. Due to this configuration, control of the center-articulated vehicle becomes a challenge because a steering angle has a direct influence on the heading error for both parts of the vehicle and, hence, small mistakes in turning can cause large path errors [36]. Since it is working off-road, it becomes more difficult due to the rough terrain of the land. There are limited control methods for center-articulated vehicles compared to traditional vehicles [35,36]. Hence, more work should be done to improve path following of the center-articulated vehicle in real field conditions [36]. Additionally, center-articulated vehicles have the potential to be used in cotton fields because they have high traction and can be small but able to pass over high-density cotton fields easily, compared to other similar sized vehicles. In this paper, a control method to improve the path following of the center-articulated vehicles is proposed, developed, and evaluated in a real field.

In this study, two IMUs, a high precision potentiometer, two encoders, a low-cost single-frequency RTK-GNSS, and the sensor fusion algorithm, Extended Kalman Filter (EKF), were utilized to perform the autonomous localization and navigation of the robot. Proportional control and a modified pure pursuit algorithm were implemented to perform autonomous cotton row following for a MPR 

The development and performance of the autonomous navigation of an MPR are presented in this paper. Autonomous means the rover can navigate itself along cotton rows without the intervention of human subjects and without destroying plants or cotton bolls. High precision is required to achieve acceptable navigation without causing an economic reduction in yield. Therefore, in this study, we present two objectives:

1. Development of the navigation system of the autonomous center-articulated hydrostatic transmission MPR.
2. Evaluation of the navigation of the autonomous center-articulated hydrostatic drive MPR in a cotton field.

## 2. Materials and Methods

### 2.1. Robot Components and System Setup

**Figure 2:**
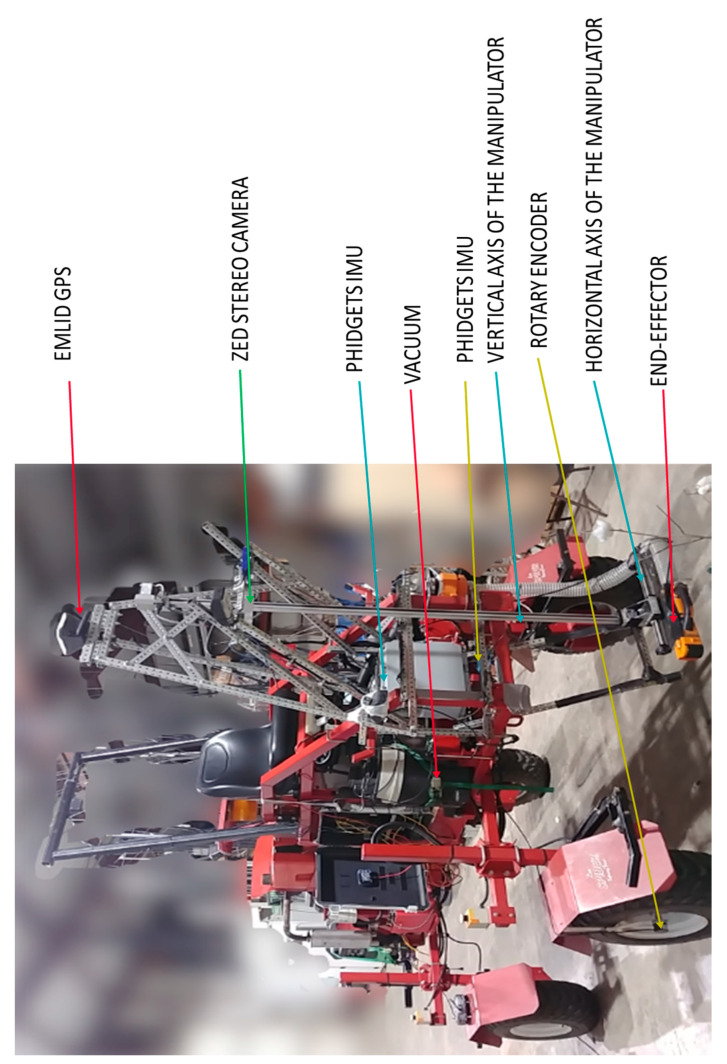
The red research rover with manipulator and sensors attached in front of the rover implemented for this study.

The rover (Figure 2) was a custom-built four-wheel center-articulated robot (West Texas Lee Corp., Lubbock, Texas). The rover was 340 cm long with front and back parts (divided by the center of articulation) being 145 cm and 195 cm long, respectively. The rover’s height and width could be adjusted to a maximum of 122 cm and 234 cm, respectively. The rover tires were 91 cm from the center of the vehicle. The rover was 212 cm wide, with a tire width of 30 cm. The four tires had a radius of 30.48 cm and a circumference of 191.51 cm. The rover had a ground clearance of 91 cm. The rover used seven sensors; two IMUs, a high precision potentiometer, two rotary encoders, and RTK-GNSS. Each front tire was connected to a rotary encoder (Koyo incremental (quadrature) TRDA-20R1N1024VD, Automationdirect.com, Atlanta, GA, USA). The two IMUs (Phidget Spatial Precision 3/3/3 High-Resolution model 1044_1B, Calgary, AB, Canada) were placed in front of the rover. The first IMU was placed 95 cm above the ground and 31 cm from the front of the vehicle. The second IMU was 132 cm above the ground and 46 cm from the front of the vehicle. The low-cost RTK-GNSS (USD 800) single-frequency receiver (EMLID Reach RS, Hong Kong, China) was placed 246 cm above the ground and 30 cm from the front of the vehicle. An embedded system (NVIDIA Jetson AGX Xavier development kit, Nvidia Corp., Santa Clara, CA, USA) was installed and used to control the rover navigation and read sensor data. 

All sensors except the rotary encoders and potentiometer were connected to the embedded system via a universal serial bus (USB). The encoders were connected to the rover navigation controller (Arduino Mega 2560, Arduino LLC) using four wires: signals A and B, power, and ground, so they can register the rotation of the tires by detecting the leading edge of rising square waves. A high precision potentiometer (Vishay Spectral Single Turn, Malvern, PA) was used to report the articulation angle of the vehicle by measuring the electric potential caused by the turn of the vehicle, which was correlated to the angle. The potentiometer was connected to the rover navigation controller.

ROS (Robot Operating System), which is robotics middleware used for robot software development, was implemented to connect the embedded system (Jetson Xavier) with the rover navigation controller. The robot software was developed to communicate using ROS topics. ROS topics were named buses that the nodes (embedded system and navigation controller) used to exchange messages [37]. The sensors connected to the embedded system published the updates that were utilized by both the embedded system and navigation controller. The topics were set to communicate so that they did not know the other nodes they were communicating with [37,38]. The rover navigation controller received a signal from the embedded computer to control the rover movement, articulation, and engine throttling. All four wheels of the rover were mounted to hydraulic motors (Parker 2090B 238 cc/rev, Parker Hannifin Corp, Mayfield Heights, OH, USA) that had their rotation controlled using a linear actuator to the swashplate lever of a 14.1 cc/rev axial-piston variable rate pump (OilGear, Milwaukee, WI, USA). The swashplate angle was controlled by the rover controller that determined the placement of the linear electric servo (Robotzone HDA4, Servocity, Winfield, KS) that had a maximum movement of approximately 10.16 cm. The left/right articulation was controlled through a 4-port 3-way open-center directional control valve (DCV) connected to two 1.75 in hydraulic cylinders (LD15012014H-A, West Texas Lee Corp., Lubbock, TX, USA) and powered by a 0.45 cc/rev fixed displacement pump (Bucher Hydraulics, Klettgau, Germany) in tandem with the variable rate pump. The DCV provided hydraulic fluid to hydraulic cylinders that controlled the rover’s articulation. The rover could turn a maximum of 45 degrees with a wheelbase of 190 cm. The engine throttle was connected to an onboard Kohler Command 20HP engine (CH20S, Kohler Co., Kohler, WI, USA) with a maximum of 2500 RPM and powered the tandem variable- and fixed-rate pumps. The speed of the engine can be adjusted to higher or lower RPMs using the servo connected to the carburetor linkage that controls the amount of air into the engine cylinders. The throttle was set at approximately 2200 RPM. The front tires were connected to a rotary encoder to provide feedback on the movement of the rover along the crop rows. Left/right articulation was controlled by using relays connected to the DCV.

### 2.2. Real-Time Kinematic GNSS and Network Transport of Radio Technical Commission for Maritime Services (RTCM) via Internet Protocol (NTRIP)

**Figure 3:**
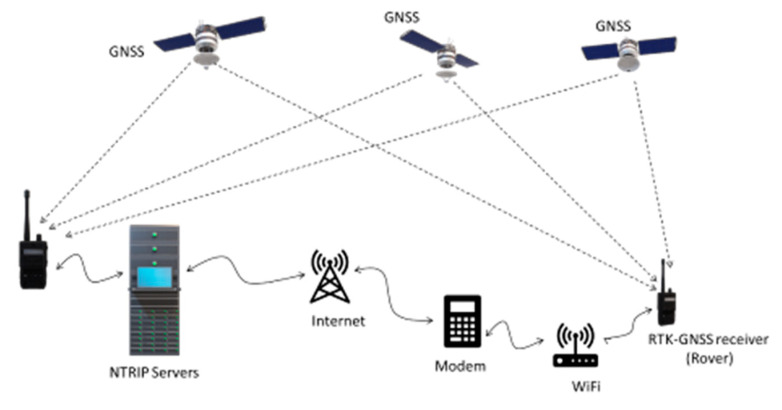
Context diagram of the Network Transport of RTCM via Internet Protocol (NTRIP).

The RTK-GNSS receiver used to acquire the global position of the rover used an NTRIP provider (eGPS Solutions, Norcross, GA, USA) to obtain differential correction through the internet using a Verizon modem (Inseego Jetpack MiFi 8800L, Verizon Wireless, New York, NY, USA) (Figure 3). The GNSS correction signal was obtained using the NTRIP signal with a mounting point within 3 kilometers of the test plot and downloaded to the RTK-GNSS through a Verizon Hotspot and wireless signal. NTRIP servers received the message from the base RTK-GNSS receivers connected to it. A data plan subscription was required to use the modem to acquire data through the internet from the eGPS base station network instead of using a local base station. The service to our NTRIP provider was registered, and a username, password, and I.P. address (mount point) to connect to the NTRIP provider via Internet Protocol were provided. Using NTRIP was advantageous because GNSS corrections were acquired without the need to set up a base station.

### 2.3. Calibration of the Potentiometer, IMUs, and Encoders

**Figure 4:**
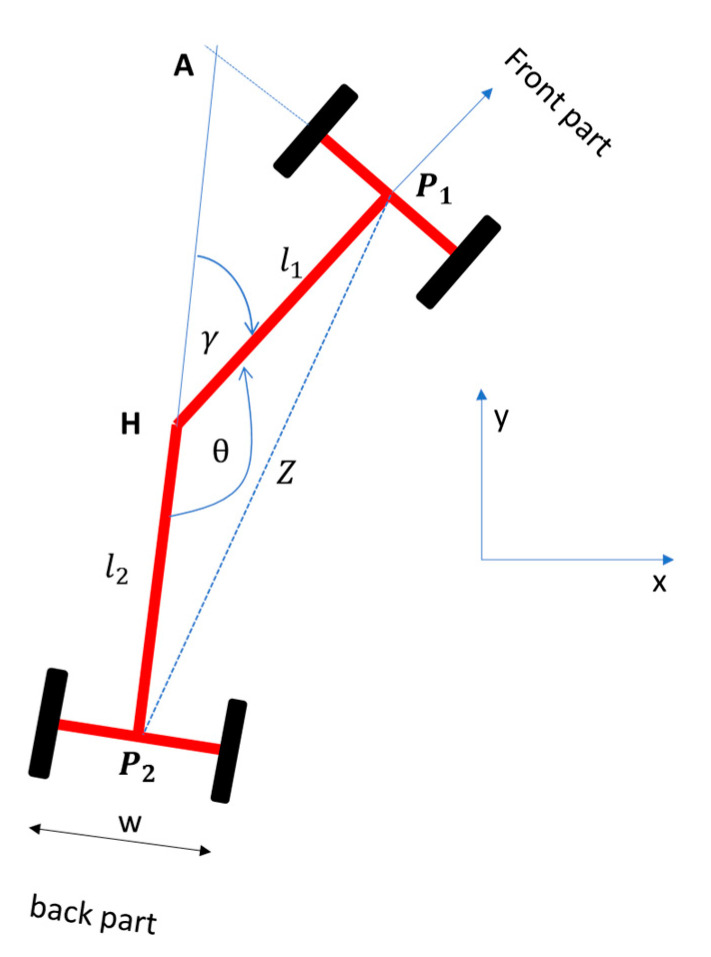
Potentiometer Calibration. Potentiometer calibration involves measurements of the voltage reported by the potentiometer in relation to the changing angle θ of the rover when turning left or right. Assume all the points are in a cartesian coordinate system.

The potentiometer (at point H in Figure 4) measured the articulation angle. Figure 4 shows the aerial view of the right turning center articulated rover. When the rover was straight, P_1_ was parallel to P_2_ and the potentiometer digital signal read 493. P_1_ is the center point between front tires, while P_2_ is the cnter point between the rear tires. The distance between P_1_ and P_2_ is Z. The length of l_1_ and l_2_ was 0.91 m each. To obtain the articulation angle γ, manual measurements were made and applied using the Cosine rule progression below (Equation (1));

(1) $$Z^{2}={\;l}_{1}{}^{2}+{\;l}_{2}{}^{2}+2\times l_{1}\times l_{2}\times \cos\;\theta$$

$$\theta =\cos^{-1}\left(\frac{l_{1}{}^{2}+l_{2}{}^{2}-Z^{2}}{2\times l_{1}{}^{2}\times l_{2}{}^{2}}\right)$$

$$\gamma =\pi -\cos^{-1}\left(\frac{l_{1}{}^{2}+l_{2}{}^{2}-Z^{2}}{2\times l_{1}{}^{2}\times l_{2}{}^{2}}\right)$$

$$\gamma \;=\pi \;-{\;\cos}^{-1}\left(\frac{0.91^{2}+0.91^{2}-{\;Z}^{2}}{2\times 0.91^{2}\times 0.91^{2}}\right)$$

**Figure 5:**
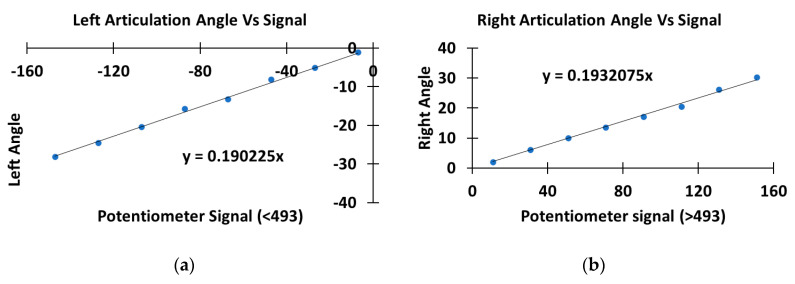
The calibration of the potentiometer and articulation angle. The left image (**a**) presents the relationship of the left articulation angle versus the potentiometer signal. The left image (**b**) shows the relationship of the right articulation angle versus the potentiometer signal.

To calibrate the angle, the rover was turned left or right. The angle was measured by the potentiometer, which was digitized with a 10-bit ADC, and the signal values ranged from 0 to 1023. The angle was recorded when turning left and right in 20 digital signal intervals from 493 (Figure 5a,b). Assume 493 as the center position and going left is negative while going right is positive. The angle γ was plotted together with the potentiometer signal (Figure 5). The potentiometer signal decreased when turning left and increased when turning right. The Equation obtained from the plots for the left was y = 0.190225x, and for the right was y = 0.1932075x (Figure 5). The Equations were implemented in Algorithm 1 at lines 15 to 19. The left/right Equations were slightly different due to potentiometer errors, human errors, and the slight misalignment of the vehicle.
**Algorithm 1:** Proportional control of the articulation angle. **Input:** Angle reported by the high precision potentiometer γ_k_ , target angle γ_k+1_ and threshold E_t_ **Output:** p which is equal to Kp* (γ_k+1_ - γ_k_)1:Gain Kp is equal to 12:p <– Kp * (γk+1 – γk)3:WHILE p > Et 4: Declare and assign 0 to increment i 5: Declare and assign 0 to temp6: WHILE i < 207:   Delay for one microsecond8:   Read the analog signal from the high precision potentiometer γk 9:   temp add the γk to temp 10:   Increment i11: }12: Get the average temp13: Assign temp to γk+114: IF temp > analog signal 493 15:   γk+1 = (temp-493) * 0.1932075;16: ELSE17:   γk+1 = (temp-493)*0.190225;18: END IF19: p <- Kp * (γk+1 - γk)20: IF p > -Et21:   Set the left relay HIGH22:   Set the right relay LOW23: ELSE IF –p < Et24:   Set the left relay LOW25:   Set the right relay HIGH26: ELSE27:   Set the left relay LOW28:   Set the right relay LOW29: END IF30:END WHILE31:Return all the error 

Both IMUs were calibrated as advised by the manufacturer’s users guide (Phidgets Inc., Calgary, CA, USA). The magnetic error correction was done by the compass calibrator software downloaded from the Phidgets website. The two IMUs were placed at two different locations on the vehicle, as described in the section “Robot components and System Setup.” The IMU was calibrated by connecting the IMU to the embedded computer, which had the Phidget compass calibration program installed. The magnetic field estimated value for Tifton, Georgia, was 0.47459 T obtained online from the NOAA website (http://www.ngdc.noaa.gov/geomag-web/#igrfwmm). After entering the magnetic field value, the program was started, and the rover was driven in a circle behind the Engineering Annex fields (31.475340N, 83.528968W) in Tifton, Georgia, to generate the calibrated compass parameters. After the calibration, the IMUs were then used for localization and navigation experiments. 

Encoder calibration was conducted by finding the circumference of the rover wheels and then converting the signal of the encoder to distance for each encoder count. The rotary encoders used a 10-bit Analog-to-digital converter. To make sure that the encoders were accurately calibrated, the tires of the rover were rotated 360°, and the count of the encoders increased from 0 to 1023. Since the circumference of the tire was 1915.1 mm, the distance per count (resolution) was 1915.1/1024 = 1.87 mm. 

### 2.4. Robot Navigation Systems

**Figure 6:**
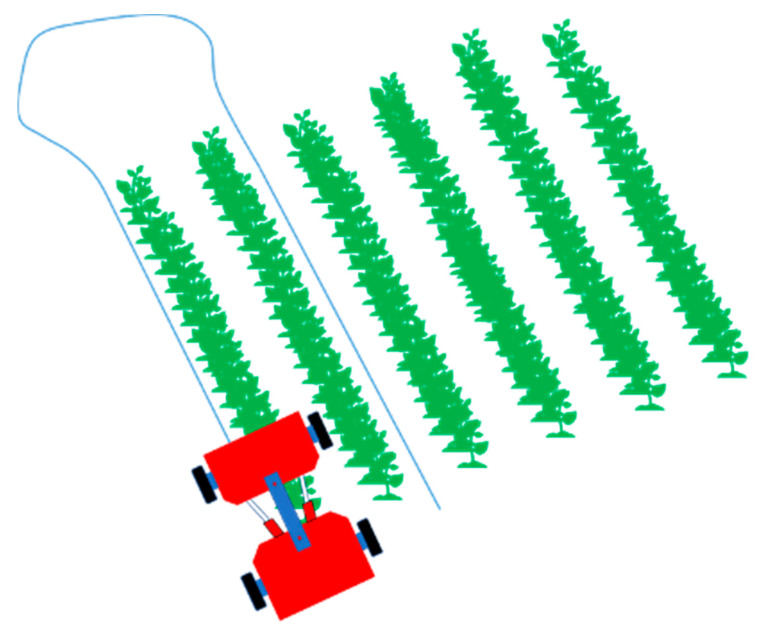
The rover driving along the cotton rows. Blue line is the path recorded by the rover after finishing one lap.

The navigation system consisted of the embedded development kit and the rover navigation controller. The rover used two algorithms to control navigation: modified pure pursuit and Proportional control [6,24,39,40,41]. The system used a predefined path of the GNSS signal to pass over the rows. The path was obtained by recording the rover path as it was manually driven down cotton rows (Figure 6). The predefined path was then used by the rover to navigate autonomously.

**Figure 7:**
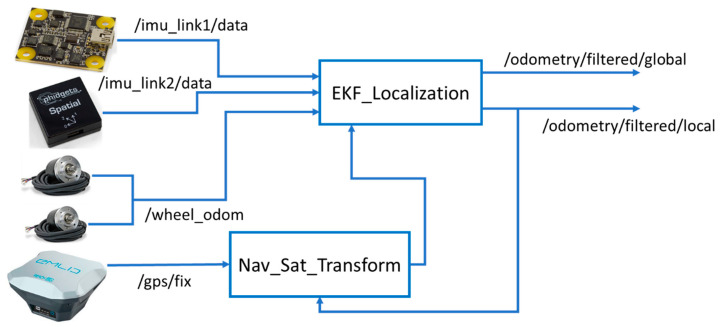
Simultaneous localization and navigation of the rover using dual Extended Kalman Filter (dual EKF).

Since the rover used six sensors (two IMUs, potentiometer, two encoders, and RTK-GNSS) to navigate (Figure 7), the Extended Kalman Filter was implemented for simultaneous localization and navigation [25,42,43,44]. Sensor fusion was achieved by using the open-source ROS library “Robot localization,” which provided sensor fusion and nonlinear state estimation for IMUs, encoders, and GNSS. The IMUs published two ROS topics (imu_link1/data and imu_link2/data), encoders published wheel odometry (/wheel_odom), and RTK-GNSS published /gps/fix signal [43]. The EKF localization (Figure 7) used the nav_sat_transform library to integrate fixed data from the RTK-GNSS [44]. Basically, "navsat_transform_node" required three sources of information: The robot’s current pose estimate in its world frame, an earth-referenced heading, and a geographic coordinate expressed as a latitude/longitude pair (with optional altitude) (http://docs.ros.org/).

**Figure 8:**
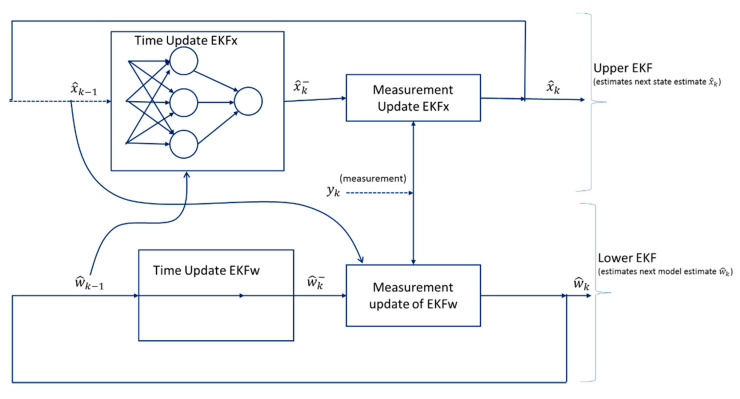
The dual extended Kalman filter. The method utilizes two concurrently running EKFs. State estimates are done by the top EKF using ${\hat{w}}_{k-1}$ for the time update, while weight estimates are generated using the bottom EKF that does measurement updates using ${\hat{x}}_{k-1}$.

EKF localization was implemented (Figure 8) as a dual EKF method that involved running two EKF’s concurrently. The state and model could be estimated from the noisy observations of the IMU, wheel odometry, and GPS fixed signal.

(2) $$x_{k+1}=F\left(x_{k},u_{k},w\right)+{\;v}_{k}$$

$$y_{k}=H\left(x_{k},w\right)+{\;n}_{k}$$

We considered the nonlinear problem in which system states (x_k_) and model parameters (w) were simultaneously estimated from the observed noisy signals (y_k_). The process noise (v_k_) drives the dynamical system, while the observed exogenous input noises (v_k_) and observation noise (n_k_) were obtained (Equation (2)). In Figure 8, two EKFs were set to run concurrently using current model estimates w_k_; an EKF state filter calculated the new state in every step [42]. The lower EKF, which takes the previous state estimate as the input and output next state, estimates the next state using the current model estimate ${\hat{w}}_{k-1},$ while the upper EKF estimates the weight using the current state estimate ${\hat{x}}_{k-1}$ Then, it calculated the fresh weights of the current state estimate x_k_. The model structures **F** and **H** are multilayer neural networks, while **w** are the weights [42]. In this case, the model used IMUs and encoders to generate localization estimates and then added RTK-GNSS to make global localization estimates of the rover position.

After getting the state estimates of the rover, a modified pure pursuit and PID were used to control the rover’s navigation.

### 2.5. Modified Pure Pursuit

Pure pursuit (P.P.) is a technique that computes the current vehicle position relative to a goal and then determines the curvature that would bring the vehicle back to the predefined or designated path. P.P. chooses the goal that is some distance in front of the rover. It looks ahead and determines the articulation of the tires to get into the path. The look-ahead distance changes depending on the curvature of the path and speed of the vehicle.

The rover achieved pure pursuit tracking by following six steps; determine the current location of the rover, find the path point closest to the rover, find the goal location, transform the goal location to rover coordinates, calculate the curvature, and request the rover to set the articulation to that curvature and then update the vehicle’s position [24,39].

**Figure 9:**
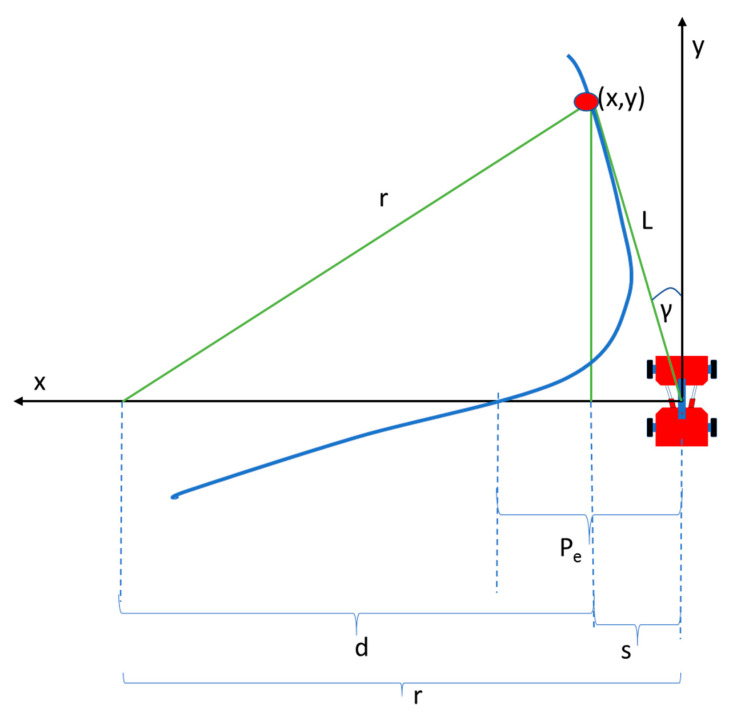
The geometry of the Pure Pursuit algorithm. Blueline is the designated path to pursue while red point (x,y) is the goal point. Black lines represent cartesian coordinates axis (y and x) while the green lines show the geometry of turning.

Figure 9 illustrates the center-articulated rover with all the sensors at the front, including GNSS steering to pursue the point (x,y) (goal point) at a distance L. L is also known as look-ahead distance. The radius of turning is r, while d is the horizontal distance of the goal from the center of the turning circle, and s is the horizontal distance of the rover to the goal. The articulation angle is γ, while Pe is the Path error. At the same time, s, which is equal to x, is the relative distance of the rover to the goal point. 

x + d = r x = s
x^2^ + y^2^ = L^2^ (3)

Using the relationship of x, y, r, and L in Figure 9, the curvature C can be derived. If x+d = r, and d^2^ + y^2^ = r^2^, r can be found by computing its relationship with x, y coordinates, r = (x^2^ + y^2^)/(2x) [6]. Then, the turning radius r becomes:

(4) $$r\;=\frac{L^{2}}{2x}$$

Hence, the curvature **C,** which is 1/r, is given as:

(5) $$C\;=\frac{2x}{L^{2}}$$

**Figure 10:**
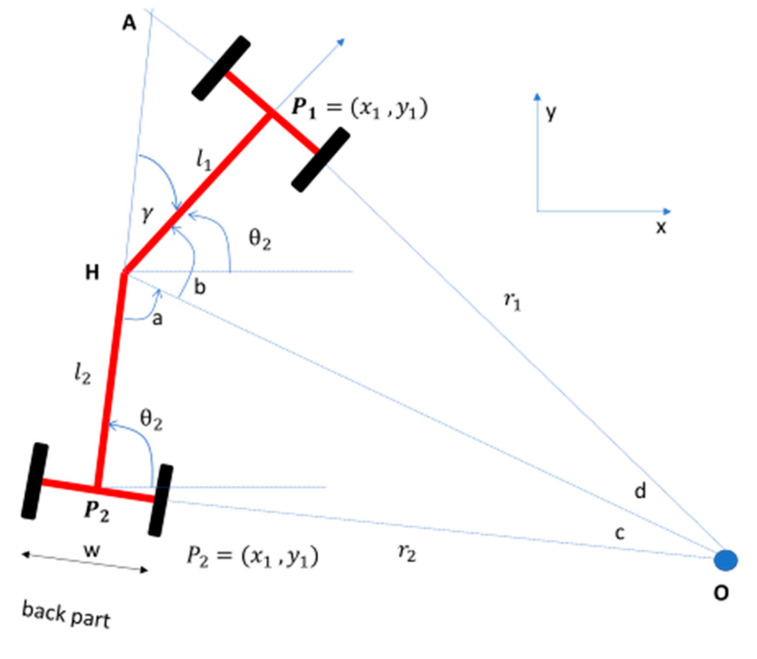
The geometry of the center-articulated rover while turning. **r**_1_ is the distance from the origin of the turning circle O [45].

Consider Figure 10, which shows the geometry of the turning rover [45]. 

At the point H, a + b + γ = 180°. So, b + d = 90° and a + c = 9°. Therefore, by substituting constants makes γ = c + d. γ is the articulation angle while θ is the heading angle.

Now, consider the right-triangle ∆OP_2_A,

(6) $$l_{2}+\frac{l_{1}}{\cos\;\gamma }={\;r}_{2}\;\tan\;\gamma$$

$$\left(r_{1}+{\;l}_{1}\;\tan\;\gamma \right)\times \sin\;\gamma \;={\;l}_{2}+\frac{l_{1}}{\cos\;\gamma }$$

Then, by simplifying Equation (6), the turning radii, r_1_ and r_2_, can be found:

(7) $$r_{1}=\frac{l_{1}\;\cos\;\gamma \;+{\;l}_{2}}{\sin\;\gamma }$$

$$r_{2}=\frac{l_{2}\;\cos\;\gamma \;+{\;l}_{1}}{\sin\;\gamma }$$

Since the rover was an approximately symmetric vehicle, the distance from the center (L_f_) to back tires or front tires was 91 cm. hence, we can simplify the radii Equation above,

(8) $$r\;={\;r}_{1}={\;r}_{2}=\frac{L_{f}\;\cos\;\gamma \;+{\;L}_{f}}{\sin\;\gamma }$$

Then, using trigonometry rules, sin 2a = 2sin a cos a and cos 2a = 2cos^2^a–1

$$\frac{r}{L_{f}}=\frac{\cos\;\gamma \;+1}{\sin\;\gamma }=\frac{2{\;\cos}^{2}\frac{\gamma }{2}}{2\;\sin\frac{\gamma }{2}\;\cos\frac{\gamma }{2}}$$

Hence, by simplifying the trigonometry,

(9) $$\tan\;\gamma \;=\frac{L_{f}}{r}$$

Then, insert Equation (4), which makes:

(10) $$\tan\;\gamma \;=2\times {\;L}_{f}\times \left(\frac{x}{L}\right)\frac{1}{L}$$

$$\tan\;\gamma \;=2\times {\;L}_{f}\times \left(\sin\;\theta \right)\frac{1}{L}$$

$$\gamma \;=2\times {\;\tan}^{-1}\left(\frac{2\times {\;L}_{f}\times \sin\;\theta }{L}\right)$$

So, Equation (10) shows the relationship of articulation angle γ to look-ahead distance L, half-length of the rover L_f_, and heading angle θ. Consider that the vehicle at state (x_k_, y_k_) is articulating to the next goal (x_k+1_, y_k+1_). Next, the heading angle θ_k+1_in relation to the current heading angle θ_k_ can be found by:

(11) $$\theta_{k+1}=\theta_{k}-{\;\tan}^{-1}\frac{x_{k+1}-{\;x}_{k}}{y_{k+1}-{\;y}_{k}}$$

Because the rover has a slow turning action when moving, the horizontal distance of the vehicle from the goal should be increased by a factor “K” (See Equation (12)). The closest distance of the rover to the path is the path error (P_e_). P_e_ is found by calculating the perpendicular distance of the vehicle to the designated path. The next position of the path (x_k+1_) was modified by adding the factor K multiplied by P_e_, becoming the modified Pure Pursuit algorithm of the center-articulated rover (Equation (12)). The value K is a rover-dependent factor that should be obtained by testing and experimentation to achieve the best path tracking.

(12) $$x_{k+1}={\;x}_{k+1}+\;K\;\times {\;P}_{e}$$

### 2.6. Proportional Control of the Articulation Angle

**Figure 11:**
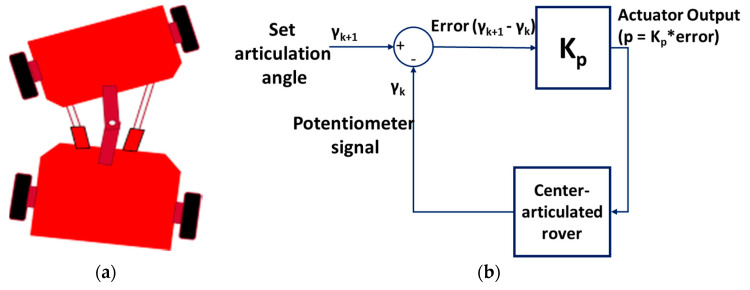
Proportional control of the articulation angle. (**a**) The rover when turning left; (**b**) the proportional control to achieve a targeted articulation angle.

The rover turned to the target articulation angle γ, as described in Equation (10) by using proportional control. The current angle γ_k_ and required target angle γ_k+1_ was used to find the error that was used to control the movement (Figure 11). The gain, Kp, used was set to 1. The articulation angle was controlled by hydraulic cylinder linear actuators. The actuators were connected to two relays. Proportional control was used since the actuators were only controlled by on/off relays, which limited the ability to control actuator speed. The two relays were connected to the navigation controller digital pin 7 for the left actuator and digital pin 8 for the right actuator. The relays used to control the linear actuator were Single Pole Double Throw (SPDT) relays. SPDT relays provided a capability to control the linear actuators by switching into three different connections: normally closed, normally open, and common [46]. So, if the rover turned left, the left actuator retracted while the right actuator extended until a desired left articulation angle was achieved. Additionally, if the rover turned right, the right actuator retracted while the left actuator extended until a desired right articulation angle was achieved. It means that the circuit opened for the right actuator and closed for the left one. When the angle required was attained, it switched to common. 

### 2.7. Proportional Control of the Speed of the Rover

**Figure 12:**
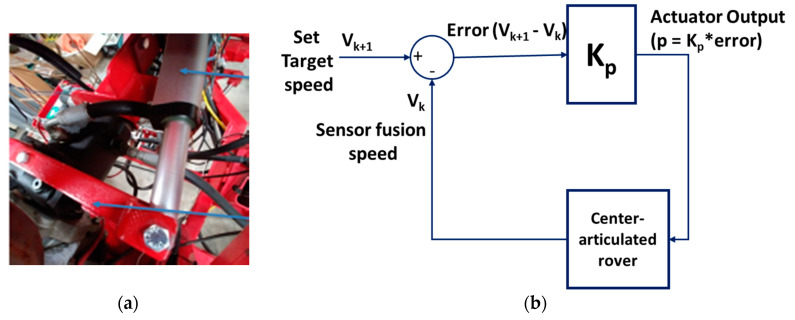
(**a**) The arrow points to the red arm that controls the swashplate angle. Another arrow points to the grey linear actuator controlled by the navigation controller (**b**) The proportional control diagram of the speed of the rover.

The speed of the rover was controlled by using the proportional controller (Figure 12). The controller had a gain of 22 and a target speed of 1.2 m/s. A PID controller was not implemented because we were not targeting precise speed control; hence, proportional control was enough. The controller calculated error from the difference between target speed and the speed estimated by the EKF from encoders, IMU, and GNSS readings. The acceleration of the rover was controlled by extending and retracting the linear actuator, which sets the swashplate angle. By changing the angle of the swashplate, hydraulic fluid flow rate to the hydraulic motors, turning the wheels was changed and effectively changed the speed of the rover. The vehicle remained stationary when the angle was set at 90°. When the angle of the swashplate changed from 90° to 60°, the vehicle moved backwards while increasing the speed to the minimum backwards speed. Additionally, when the angle of the swashplate changed from 90° to 120°, the vehicle moved forward with the increase in the maximum forward speed. However, the change in actuator movement required the swashplate angle to be more than 108° to move forward, or less than 80° to move backward, created by an inherent deadband in the pump performance. The deadband was caused by wear (leakage) in the hydraulic system and the mechanical compliance of connectors. The neutral position was held until the actuator extended or retracted by more than 2.5 cm. 

### 2.8. Waypoints Collection and Cubic Spline Interpolation of the Waypoints

The rover was driven to obtain the waypoints at the UGA grounds behind the engineering annex located at (31.475340 N, 83.528968 W) and the Entomology farm (31.472985 N, 83.531228 W) near Bunny Run Rd. in Tifton Georgia. The rover recorded the waypoints using RTK-GNSS at the rate of 5 Hz. Since the rate 5 Hz provided very few data points, the algorithm to interpolate the points using a cubic spline interpolation method was developed. The points were changed to UTM data points. Both fields were located at Zone 17R. 

Cubic spline interpolation was done by assuming the data points were connected by a line whose Equation was a polynomial degree of three [47]. It was assumed that the datapoints given were [x_i_, y_i_], and no two points xi were equal to each other, and the x_i_ was in sequence such that x_0_ < x_1_ < x_2_ < …< x_n_. The Spline function S(x_i_) = y_i_ . For each subinterval [x_i-1_ < x < x_i_], the cubic function is given as $C_{i}=a_{i}+b_{i}x+c_{i}x^{2}+d_{i}x^{3}$. So, for every subinterval, the Spline function S(x) can be assumed as:

$$S\left(x\right)=\{\begin{array}{c} C_{1}\left(x\right),\;\;\;\;\;\;\;\;x_{0}<x<x_{1}\\ C_{2}\left(x\right),\;\;\;\;\;\;\;\;\;x_{1}<x<x_{2}\\ \;\;\;\;\;\vdots \\ C_{i}\left(x\right),\;\;\;\;\;\;\;\;\;\;x_{i-1}<x<x_{i}\\ \;\;\;\;\;\vdots \\ C_{n}\left(x\right),\;\;\;\;\;\;\;\;\;x_{n-1}<x<x_{n} \end{array}$$

Algorithm 2 is used to calculate the value of a to calculate the values of a_i_, b_i_, c_i_ and d_i_ for every interval of the dataset. More values make it easy to calculate the relative position of the rover to the target path perpendicularly.
**Algorithm 2:** Cubic Spline Algorithm to estimate subinterval of UTM waypoints data intervals. **Input:** x_0_, x_1_, x_2_, …, x_n_; a_0_ = f(x_0_), a_1_ = f(x_1_), a_2_ = f(x_2_), ….. a_n_ = f(x_n_) **Output:** ai, bi, ci, di for j = 0,1,2,…..,n-11:Assign P_0_ = 02:Assign Q_0_ = 03:Assign R_0_ = 04:FOR j = 0 TO n-15:   Set the interval difference hi <- x_i+1_ - x_i_6:   Set α_1_ = (3/h_j_)*(a_j+1_ – a_j_) – (3*(a_j_ – a_j-1_)/h_j-1_)7:END FOR8:FOR j = 1 TO n-19:   P_j_ = 2*(x_j+1_ – x_j-1_) – h_j-1_Q_j-1_10:   Q_j_ = h_j_ / L_j_11:   R_j_ = (α_j_ – h_j -1_*R_j-1_)/P_j_12:END FOR13:Assign P_n_ = 114:Assign R_n_ = 015:Assign c_n_ = 016:FOR i = n-1 TO 017://get the remaining values of the cubic spline b,c, and d18:   c_i_ = Z_i_ – Q_i_ * c_i+1_19:   b_i_ = (a_i+1_ – a_i_)/h_i_ – h_i_*(c_i+1_ + 2*c_i_)/320:   d_i_ = (c_i+1_ – c_i_)/3*h_i_21:Return all the values of ai, bi, ci, di 

### 2.9. Preliminary Experiment

Preliminary experiments were conducted on 10th October 2019 at the UGA grounds to study the navigation behavior of the rover, when parameters such as ROS update rates, look ahead, and path error were altered. These tests served to calibrate the system to perform well in the field. The preliminary experiment involved four tests:

1. A fast ROS rate was set to 10 Hz;
2. A short look ahead was set at 1 m;
3. Path error was set to 0 which means K x P_e_ = 0;
4. An optimal condition was set (long look-ahead is 3 m, path error is 1.5 times path error and slow ROS rate at 1 Hz).

The experiment was conducted by setting the rover to follow the prescribed path. The predefined path was obtained by moving the rover manually and collecting the GNSS waypoints. Later, the rover was set to autonomous mode to follow the pre-planned path so that behavior and characteristics could be observed and tuned.

### 2.10. Field Experiment

**Figure 13:**
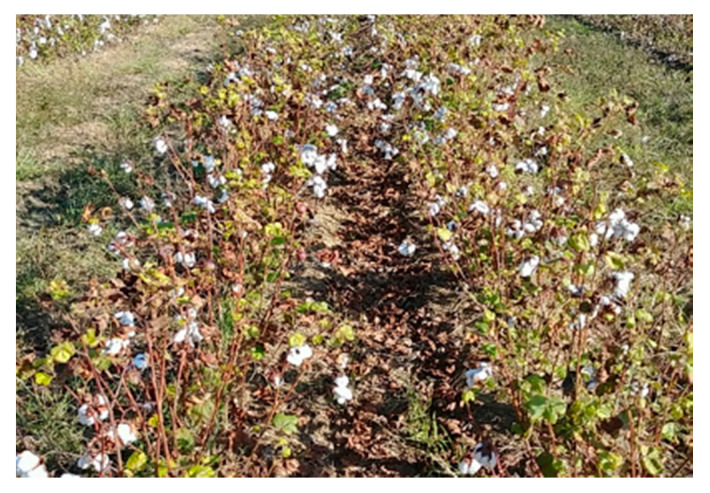
The appearance of the cotton at the time of the field experiment.

The field experiment was conducted at the Horticulture hill farm (31.472985N, 83.531228W) near Bunny Run Road in Tifton, Georgia, after establishing the calibrated parameters of the rover. The field (Figure 13) was planted on 19th June 2019 using a tractor (Massey Fergurson MF2635 tractor, AGCO, Duluth, GA, USA) and a 2-row planter (Monosem planter, Monosem Inc, Edwardsville, KS, USA). The cotton seeds (Delta DP1851B3XF, Delta & Pine Land Company of Mississippi, Scott, MS, USA) were planted every two-rows and skipped two rows. The rows were 36-inch (91.44 cm) wide, and the seed spacing was 4-inch (10.16 cm). The cotton field was undefoliated, and most of the cotton bolls were open already at the time of the experiment. Three tests were conducted for navigation on 18.5 m rows on 21st October 2019. The path was obtained by driving the rover over one of the two rows of cotton plants and collecting the waypoints (Figure 6). The experiments were conducted by setting the rover to follow the predefined path autonomously.

## 3. Results and Discussions

### 3.1. Preliminary Experiment

**Figure 14:**
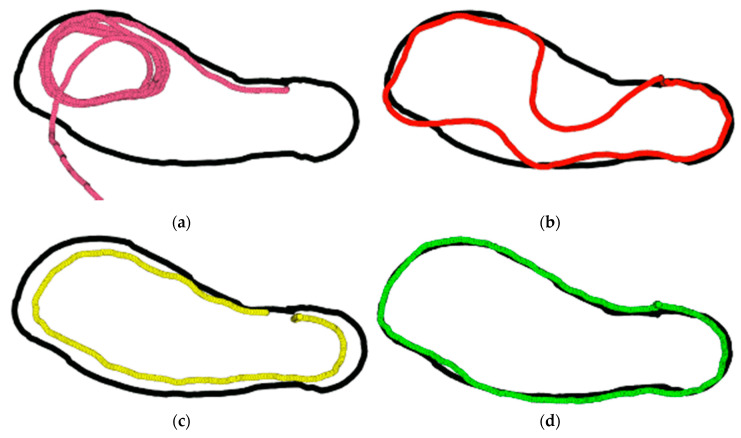
Performance of the rover when the (**a**) ROS updates were high (10 Hz), (**b**) Short look ahead distance (1 m), (**c**) no path error correction was applied and (**d**) successful path tracking when look-ahead was 3 m, K was 1.5, and ROS updates were at 1 Hz. Blacklines are the predefined waypoints, while colored lines represent the rover passes for each condition from 1 to 4.

The results of the preliminary experiments show that the rover navigation tracking was negatively affected when ROS update rates were increased, or no path error correction was applied, and when very short look-ahead was used (Figure 14). The pure pursuit algorithm has a goal to make sure that the rover regains the designated path by articulation when it loses where it was on the predefined waypoints. Fast ROS update rates affected system performance since the mechanical responses of the machine were slow compared to the update rate provided by the controller. This short time between the new input reading of the system meant that the reaction time of the vehicle to the control signal was slow, and the vehicle reaction was always lagging behind the control decision, causing the rover to lose tracking control. The ROS rate update was a significant parameter if it was not set right. The performance was obtained with short look-ahead distances. With a short look ahead distance, the rover tried to move quickly to regain the path it has lost. However, this action caused the rover to overshoot the path and oscillate along the prescribed path.

With no path error corrections (when K was Zero), the rover could never converge to the path over time. Figure 14c shows how the vehicle was not able to converge to the path, which means the error was consistently maintained. To avoid this behavior, modified pure pursuit increased the error of the system by 1.5 using Equation (12) to force the system to converge to the path. If the error was significant, the system amplified the error forcing the rover to act aggressively and quickly. When the error was small, the system acted slowly because the amplification of the error also became small (Refer to Equation (12)).

### 3.2. Field Experiments

**Figure 15:**
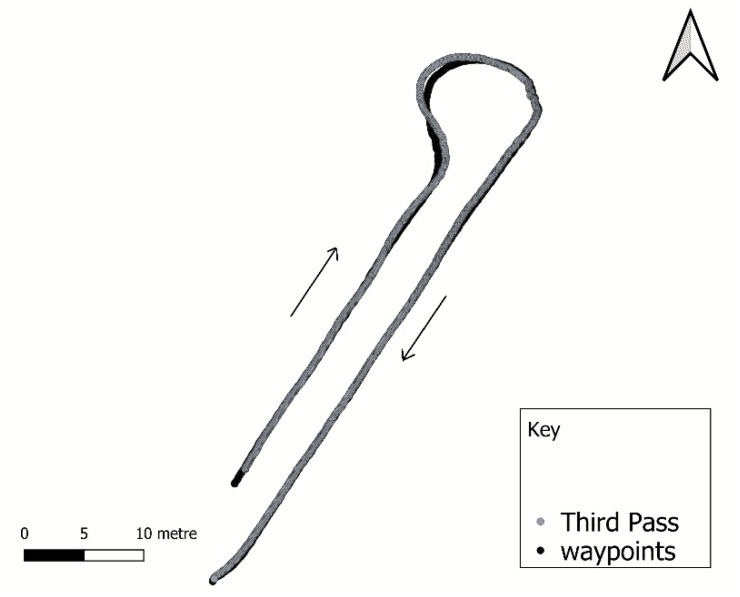
Path tracking of the prescribed path (black pass). The gray pass is the GPS generated path of the rover when following the rows using the prescribed path (black). The gray path trace is the third navigation pass experiment. The arrows show the passing direction of the rover.

Figure 15 shows the navigation path traces, as recorded by the GNSS for the third experiment. The rover performed well visually. The third pass can clearly show that the rover moves out of the path during the end-of-row turning. Contributing factors were wheel slipping as the rover attempted to turn compounded by control signal updates requiring turning too quick for the rover to respond and follow the designated path. 

**Figure 16:**
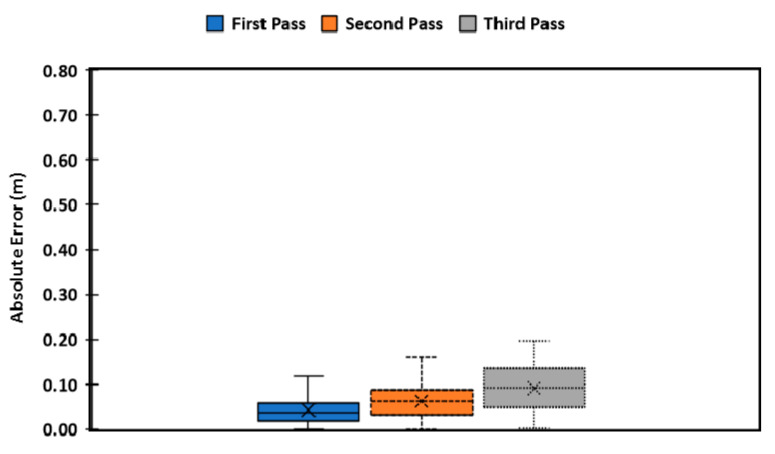
The average of absolute errors (A.E.) in the first, second, and third non-turning passes (blue, orange, and gray boxes, respectively). The boxes present the first quartile to the third quartile of A.E., while the whiskers show maximum values and minimum values of each of the passes. The “x” shows the mean absolute error (MAE) for each of the passes.

The rover was able to follow the path and return to enter the next cotton row. The absolute error distribution was determined to characterize rover navigation behavior. The rover performed well, as Figure 16 shows most of the path errors were less than 15 cm (0.15 m).

**Figure 17:**
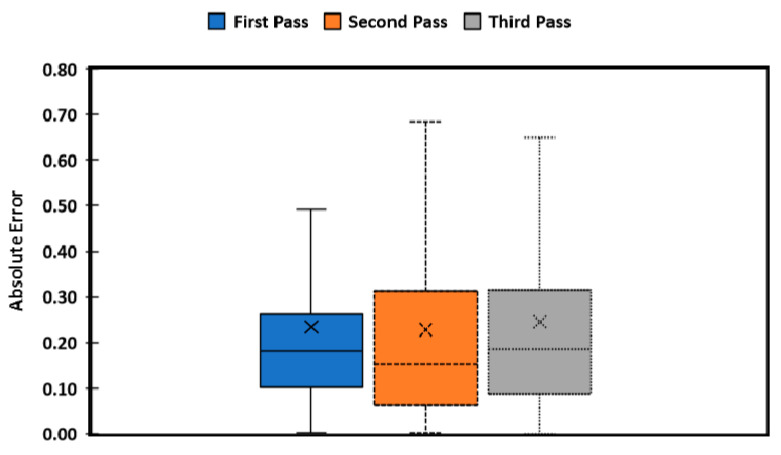
The average of absolute errors (A.E.) when turning for the first, second, and third passes (blue, orange, and gray boxes, respectively). The rover had no significant difference in turning performance. The boxes present the first quartile to the third quartile of A.E., while the whiskers show maximum values and minimum values of each of the passes. The “x” shows the mean absolute error (MAE) for each of the passes.

Figure 16 and Table 1 show that the rover performance was adequate as it was safely navigating along the rows without driving over the plants. Most of the errors were less than 10 cm from the prescribed path. The rover converged back to the predefined path when there was an error, except during the turning maneuver, which provided a significant challenge to the rover. The first and second tests were excellent, but the third one had trouble turning in the muddy end-row that caused the wheels to slide. The MAE of 0.042 m, 0.062 m and 0.091 m for the first, second, and third pass, respectively, show that the system performance was acceptable. However, the MAE was significant when turning as it ranged at 0.233 m, 0.227 m and 0.244 m for the first, second, and third pass, respectively (Figure 17). Slippage had effects only in the third pass when turning caused errors to increase to 0.115 m in the second pass. Additionally, the results show a modified simple pursuit algorithm was struggling to compensate for errors in the third pass. However, the magnitude of the errors measured when turning did not inhibit overall performance for harvesting.

## 4. Conclusions

An autonomous navigation algorithm using a modified pure-pursuit algorithm for an MPR was designed, developed, and tested in this study. The results showed that the rover could autonomously navigate safely along the rows of cotton, turn around, and enter a second row. Results from the preliminary testing and field testing showed that an affordable single-frequency RTK-GPS could be used with other sensors and a sensor fusion technique to achieve acceptable navigation accuracy. There was an increase in errors when the rover performed turns that did not impede the rover’s ability to enter the next row. As a result, the MPR can follow rows and operate autonomously to perform any number of tasks when a predefined path in GNSS coordinates is available or created. In the future, small, intelligent, multi-purpose vehicles that can autonomously navigate on the field could provide paths and prescriptions for spraying, planting, scouting, or harvesting. Small rovers would eventually need to operate in teams to cover larger acreages with rover-to-rover communication to create built-in task optimization. 

## Figures and Tables

**Table 1:** The mean absolute error and standard deviations of the three passes along the cotton rows.

Mean ± Std. Dev (m)	1st Pass	2nd Pass	3rd Pass	Overall
**1St Row**	0.048 ± 0.036	0.048 ± 0.035	0.066 ± 0.0046	0.053 ± 0.041
**Turning**	0.233 ± 0.198	0.227 ± 0.211	0.244 ± 0.211	0.235 ± 0.206
**2nd Row**	0.036 ± 0.024	0.081 ± 0.028	0.115 ± 0.043	0.070 ± 0.046
**Overall (1st and 2nd Rows)**	0.042 ± 0.032	0.062 ± 0.036	0.091 ± 0.053	0.061 ± 0.044
